# A new approach for estimating living vegetation volume based on terrestrial point cloud data

**DOI:** 10.1371/journal.pone.0221734

**Published:** 2019-08-29

**Authors:** Le Li, Changfu Liu

**Affiliations:** 1 Research Institute of Forest Ecology, Environment and Protection, Chinese Academy of Forestry, Beijing, China; 2 Key Laboratory of Forest Ecology and Environment, State Forestry and Grassland Administration, Beijing, China; Technical University in Zvolen, SLOVAKIA

## Abstract

Living vegetation volume (LVV), one of the most difficult tree parameters to calculate, is among the most important factors that indicates the biological characteristics and ecological functions of the crown. Obtaining precise LVV estimates is, however, challenging task because the irregularities of many crown shapes are difficult to capture using standard forestry field equipment. Terrestrial light detection and ranging (T-LiDAR) can be used to record the three-dimensional structures of trees. The primary branches of *Larix olgensis* and *Quercus mongolica* in the Qingyuan Experimental Station of Forest Ecology at the Chinese Academy of Sciences (CAS) were taken as the research objects. A new rapid LVV estimation method called the filling method was proposed in this paper based on a T-LiDAR point cloud. In the proposed method, the branch point clouds are divided into leaf points and wood points. We used RiSCAN PRO 1.64 to manually separate the leaf points and wood points under careful visual inspection, and calculated that leaf points and wood points accounted for 91% and 9% of the number of the point clouds of branches. Then, the equation *LVV = V*_*1*_*N* (where *N* is the number of leaf points, and *V*_*1*_ is cube size) is used to calculate LVV. When the laser transmission frequency is 300,000 points/second and the point cloud is diluted to 30% using the octree method, the point cloud can be replaced by a cube (*V*_*1*_) of 6.11 cm^3^ to fill the branch space. The results showed that good performance for this approach, the measuring accuracy for *L*. *olgensis* and *Q*. *mongolica* at the levels of α = 0.05 and α = 0.01, respectively (94.35%, 90.01% and 91.99%, 85.63%, respectively). The results suggest that the proposed method can be conveniently used to calculate the LVV of coniferous and broad-leaf species under specific scanning settings. This work is explorative because hypotheses or a theoretical framework have not been previously defined. Rather, we would like to contribute to the formation of hypotheses as a background for further studies.

## Introduction

In the 1990s, to evaluate the level of urban greening, Chinese scholars proposed the concept of “tridimensional green biomass (TGB)”, which refers to the volume of space occupied by the stems and leaves of all growing plants [1]. We describe TGB as “living vegetation volume (LVV) [2]”. The study of LVV is based on the ecological function and physiological metabolism characteristics of plant stems and leaves, which reflect the material flow, energy conversion and utilization of plants. Since branches are the main site for photosynthesis in trees, LVV can indicate the productivity of forest ecosystems and the accumulation of dry matter in trees. Estimation of the LVV is essentially the measurement of volume, which is mainly replaced by the crown volume at present. Crown volume can also be a good alternative predictor for foliage biomass [3] and potentially for the whole tree biomass. In addition, crown volume, as one of the most difficult tree parameters to calculate [4], is also among the most important factors indicating the biological characteristics and ecological functions of the crown, and it directly influences the photosynthetic efficiency of the tree, which in turn influences forest productivity [5]. Therefore, the LVV of a forest is one of the most important statistics in forest management.

Since the concept of the LVV was formally presented, much attention has been focused on LVV estimations in major cities throughout China. To estimate large-scale LVV, previous research has mainly applied remote sensing technologies [6, 7], which can be divided into the following categories: 1. simulating the stereo quantity by the plane quantity [8, 9], in which the crown breadth is used to estimate the crown height, and then the crown volume is deduced based on the aerial image; and 2. estimating the tridimensional volume by the tridimensional volume [10], in which the left and right parallaxes of two adjacent aerial photos are used to determine the vegetation height; the area of the vegetation is measured on the aerial photos; and then the LVV is calculated according to an empirical formula. Since the tridimensional structure is derived from two-dimensional remote sensing images, so the above methods perform better at high resolution than at regular resolution. Optical aerial photography does not directly provide information on 3D forest structure [11], and capturing individual tree characteristics has limitations [12].

The traditional method for calculating the volume of an individual tree crown utilizes a volume equation that includes tree height (H) and diameter at breast height (DBH) [13] and an expression of tree form or to match the regular geometry that is similar to the crown shape [10]. Measurement methods include field inventories and aerial photography interpretation, and the tree crown is simulated as a regular geometry in the measurement of the LVV. These parameters are mainly acquired from field inventories, which can be labor-intensive, time-consuming, and limited by spatial accessibility [14, 15]. Meanwhile, the crowns are randomly formed, and the difference between the regular geometry and the real shape is large. Most of these limitations of remote sensing images and the traditional method can be overcome by using LiDAR measurements. Wei *et al*. [16] used LiDAR point cloud data and cone to measure the crown volume of 45 *Sabina chinensis* and calculated the relative error of crown volumes obtained by the two methods. The results showed that the average relative error was 44.75% and the closer the crown of *S*. *chinensis* was to the standard cone, the smaller the relative error. In addition, two trees with similar crown volumes would have different spatial densities of leaves. Therefore, calculating the LVV based on the volume empirical formula would be unreliable considering the large differences between trees.

LiDAR is an active remote-sensing technology that can be used to record three-dimensional (3D) information about objects [17–20]. According to the platform, LiDAR can generally be categorized into three types: satellite-based LiDAR, airborne LiDAR, and terrestrial LiDAR (T-LiDAR) [21]. LiDAR has numerous advantages over optical remotely sensed imageries, such as high sampling rate, extensive areal coverage, ability to penetrate the top layer of the canopy, precise geolocation, and accurate ranging measurements [22]. In recent years, the use of LiDAR technology to measure the biophysical characteristics of forests has rapidly increased. Forest-related applications need different levels of detail [23]. The potential of airborne laser scanning (ALS), including laser scanning utilizing unmanned aerial vehicles (UAVs), is well known in the literature for applications at the landscape and regional scales, and several recent works and reviews provide important points of reference [24, 25]. Of all the LiDAR platforms available, T-LiDAR operates underneath the canopy; therefore, it is able to acquire dense point clouds that record detailed branch structures for both the canopy and overtopped trees in the subcanopy layers [19]. During the last few decades, most of the research on T-LiDAR in the forest environment has focused on developing automated algorithms for plot-scale forest inventories, i.e., DBH and H estimates [26, 27]. Since a detailed representation of single trees is important to forest ecosystem research, some studies have focused on modeling individual trees using LiDAR data, for example, based on circle fitting [28], cylinder fitting [29], voxel-based processing [30], tree meshing [31], and geometric fitting [26]. These studies demonstrate the potential of using T-LiDAR to characterize the woody structures of trees.

The crown volume of an individual tree can be calculated from T-LiDAR data using a voxel method [32–34] or a Delaunay triangulation algorithm [35] as its representatives. The former uses discretized cloud points in voxels to render discontinuous crown surfaces and the crown volume can be calculated by counting the effective number of voxels [36]. Such algorithms are well known for their robustness, but they are slow and suffer from discretization artifacts [35]. In addition, this method cannot completely prevent the occultation of points inside the crown by external leaves and branches and the voxel size needs to be chosen carefully because it can significantly influence the estimation accuracy of the volume [37, 38]. Any changes in sample design and/or scan setup (e.g. number of scans, size of plants, distance away from tree, etc.) could potentially affect the point cloud density and complexity and consequently the selection of voxel size [23]. The latter progressively fits discrete point clouds to a continuous triangular mesh, covering the whole surface of the crown to generate a 3D model and to extract the crown factor. This method can better restore details on the continuous surface of the object with less calculation time [39]. However, the classical Delaunay algorithm is generally sensitive to these factors (e.g., size of plants, tree characteristics and structure, point cloud density, etc.) and therefore lacks robustness. Furthermore, these methods are difficult to implement for basic forest investigators who lack a programming background. Therefore, another simple, inexpensive, rapid and reliable method for estimating LVV is required for investigators and researchers.

The general aim of this study is thus to propose a method for quickly estimating LVV with the support of terrestrial point cloud data. This paper takes the primary branches (containing leaves) of *Larix olgensis* and *Quercus mongolica* in the eastern mountainous area of Liaoning Province, China, as examples to estimate the LVV using point cloud data obtained via T-LiDAR. The core idea of the LVV estimation method is to treat each point in the leaf point cloud data as a cube; then, more space is covered by increasing the size of the cube, and when the space of the covered branch is fulfilled by the cubes, the LVV is the sum of all regular geometric volumes. Based on the assumption, the branch volume is ideally the product of the number of leaf point clouds when the cube volume is reached; therefore, the key step of this method is to find the regression relation between the number of leaf point clouds and the LVV. This work is explorative as it is quite different from existing methods, and the results will contribute to LVV estimations with point clouds.

## Datasets and methods

### Study site

Experimental sampling was implemented in the Qingyuan Forest Chinese Ecosystem Research Network (Qingyuan Forest CERN), Chinese Academy of Sciences (CAS). The Qingyuan Forest CERN (41.84°-42.86°N, 124.89°-125.95°E) was founded in 2003 by the Institute of Applied Ecology, CAS/Qing Yuan County and is located in southeastern Qingyuan Manchu Autonomous County, Liaoning Province, China. The area belongs to a continental monsoon climate that is characterized by hot and rainy summers and long cold winters. The annual average temperature is 3.9 ~ 5.4°C, the maximum temperature is 36.5°C, the lowest temperature is -37.7°C, the frost-free period is 120 ~ 125 d, and the annual precipitation is 700 ~ 850 mm. The soil is brown forest soil, and the pH value is 5.5 ~ 6.5.

The Qingyuan Forest CERN has approximately 1350 ha of experimental forest consisting of various types of natural secondary forest formed after intense disturbances to the original forests in the 1950s. This area is an extension of Changbai Mountain with a height of 500 ~ 600 m above sea level. The major plantations in the forests include Korean pine (*Pinus koraiensis*) and larch (*Larix spp*.). The typical forest vegetation belongs to Changbai Mountain flora. The regional vegetation climax is mixed broadleaved Korean pine forest.

**Fig 1:**
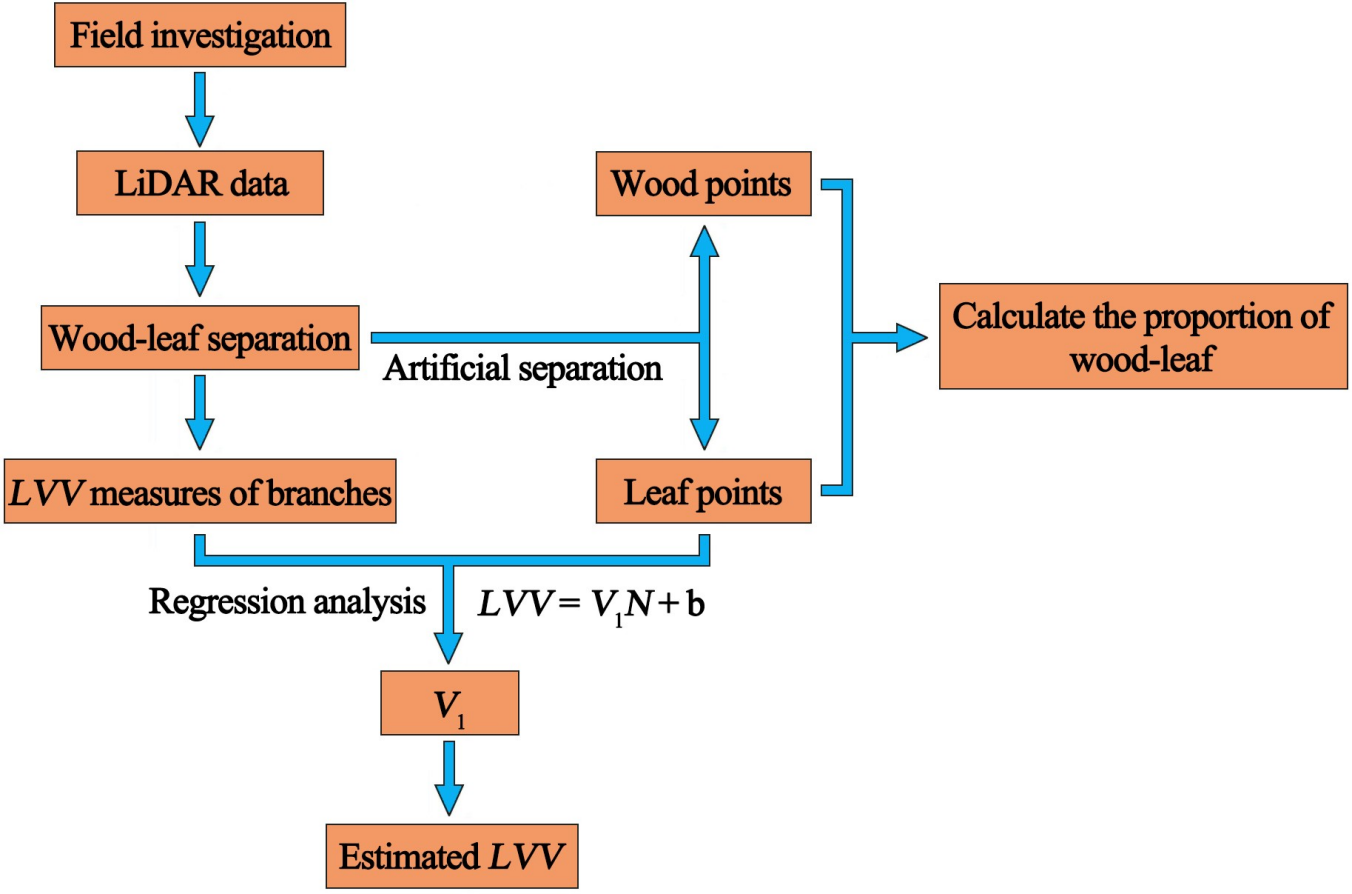
Flowchart of branch LVV estimation.

Fig 1 illustrates the LVV estimation workflow, which consists of wood-leaf separation and LVV estimation. The details are then presented.

### Sample setting and standard tree selection

Three standard sampling plots of 20 m × 20 m were selected in the experimental forests of the Qingyuan Forest CERN in September 2014. These plots were dominated by *L*. *olgensis* that were more than 50 years old, but the plots had different plantation densities. Measurements were carried out for all trees in the plots. The H, DBH, first live branch height (H1), and the crown width (CW) in four directions were recorded for each tree, and then the forest average H, DBH, CW and plant density were calculated for each plot and all trees (Table 1). A well-grown, healthy *L*. *olgensis* tree with a uniform crown and average DBH and H was selected as the standard tree in the vicinity of each standard plot. After harvesting and taking the whole plant to the experimental station, we carefully marked and cut all branches to avoid further damage to the branches and leaves. In addition to three standard woods of *L*. *olgensis*, we also had one broad-leaf tree species, *Q*. *mongolica*. Since the *Q*. *mongolica* forest was the natural secondary forest, so we only cut one primary branch of one selected well-grown *Q*. *mongolica* tree.

**Table 1:** Information on the standard plots in the *L*. *olgensis* plantation.

Plot	Average H/(m)	Average DBH/(cm)	Average CL/(m)	Average CW/(m)
A	30.71	24.85	16.34	6.57
B	23.41	28.01	12.33	7.76
C	24.44	24.05	12.17	5.42

### Analysis of branches

Each branch of one *L*. *olgensis* standard tree was numbered consecutively from the bottom stem to the crown. Overall, 174 branches of *L*. *olgensis* and 43 branches of *Q*. *mongolica* were obtained. Then, we measured the diameter (*d*) and length (*L*) of all branches (Table 2, S1 Table).

**Table 2:** Basic information of *L*. *olgensis* branches.

Branch factors	Mean	Min	Max	Std
*d*/(mm)	25	6.28	57.48	8.91
*L*/(cm)	224.5	88	480	78.83

Note: *d*: diameter of the branches, *L*: length of the branches

### Point cloud data acquisition

The point cloud data were acquired by a RIEGL VZ-1000 laser scanner system, which has a horizontal scanning angle of 360° and a vertical scanning angle of 100° (60° on and 40° below the horizontal plane) with a laser emission frequency of 300,000 points/second. The minimum scanning distance of RIEGL VZ-1000 is 2.5 m, and the maximum scanning distance is 1400 m (four scan maximum range options corresponding to point density or scan accuracy of 450 m, 950 m, 1200 m, and 1400 m). The highest point density of the point cloud, or the distance between two points, is 5–8 cm at the 100 m distance under a scan option of 450 m. At the same time, a D700 Nikon camera was mounted on the top of the RIEGL VZ-1000 to simultaneously obtain images of the scanning objects when scanning.

**Fig 2:**
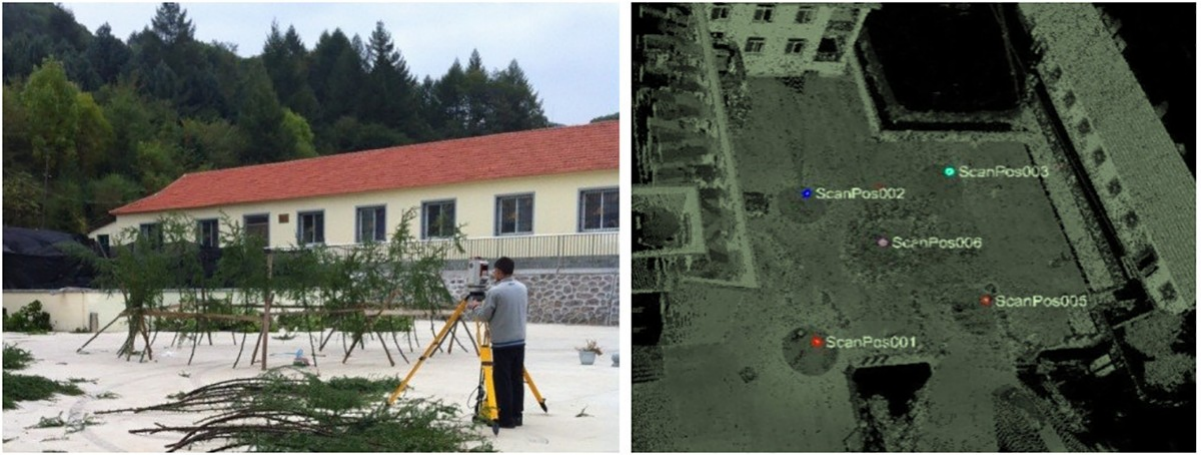
Position layout.

The scanning process was carried out in the yard of the research station, and there were no other trees nearby that would affect the scanning objectives (Fig 2). To obtain more detailed information, we made a seven-sided bracket with a height of approximately 1 m, placed the bracket upright and fixed all branches to the bracket with enough space between two branches to avoid overlaps. After fixation, the positions of the branches were photographed and recorded to identify each branch in the further point cloud analysis. Five target pieces with diameters of 5 cm were placed in locations where they were easily recognized from the scan sites, and at least three identical targets occurred in two scan positions. These target pieces were circular and reflected at high intensities; thus, they were more easily recognized as red points in the point cloud data. Therefore, these pieces can be used for co-registration among the point data from different scan positions. Five scanning positions were selected: four positions outside the bracket in four directions and one positions inside the bracket with a distance less than 10 m from the branches (Fig 2).

**Fig 3:**
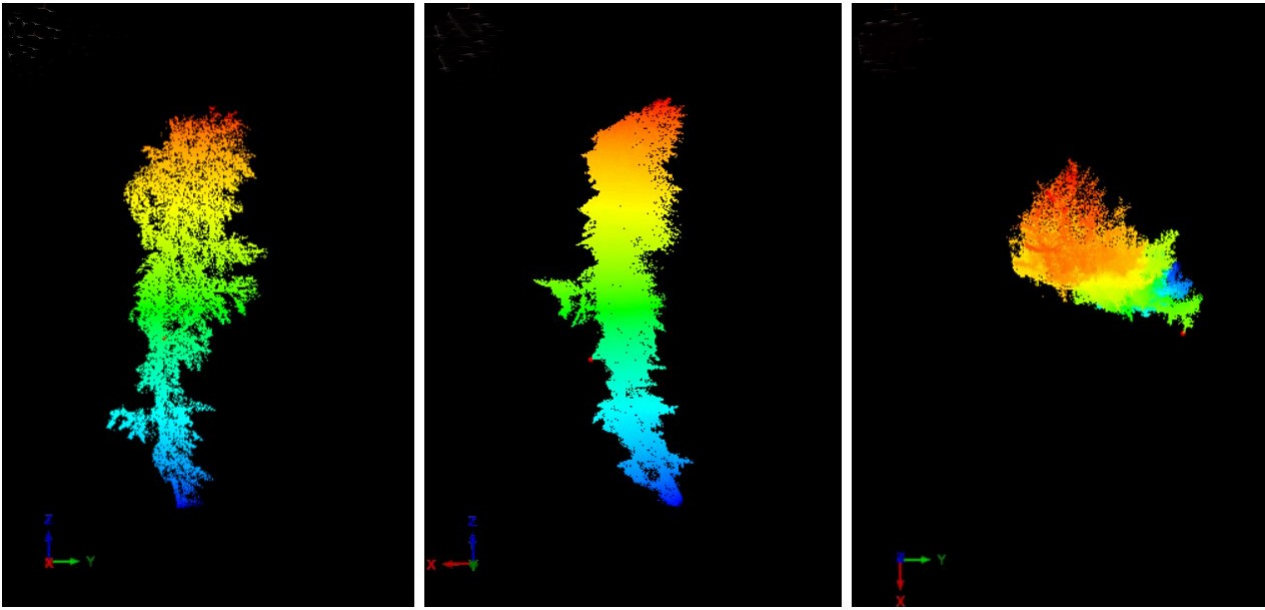
Point cloud of the *L*. *olgensis* branch (A: main view; B: side view; C: top view, 121034 points).

After scanning, the original point cloud data were preprocessed using the RiSCAN PRO1.64 software, including splicing, removing outliers and thinning. Fig 3 and S1 Fig shows the data form the *L*. *olgensis* and *Q*. *mongolica* branches point cloud.

### Wood-leaf separation

A manual method is used for wood-leaf separation, which aims to classify LiDAR points into wood and leaf components, and it is an essential prerequisite for deriving individual tree characteristics [40].

**Fig 4:**
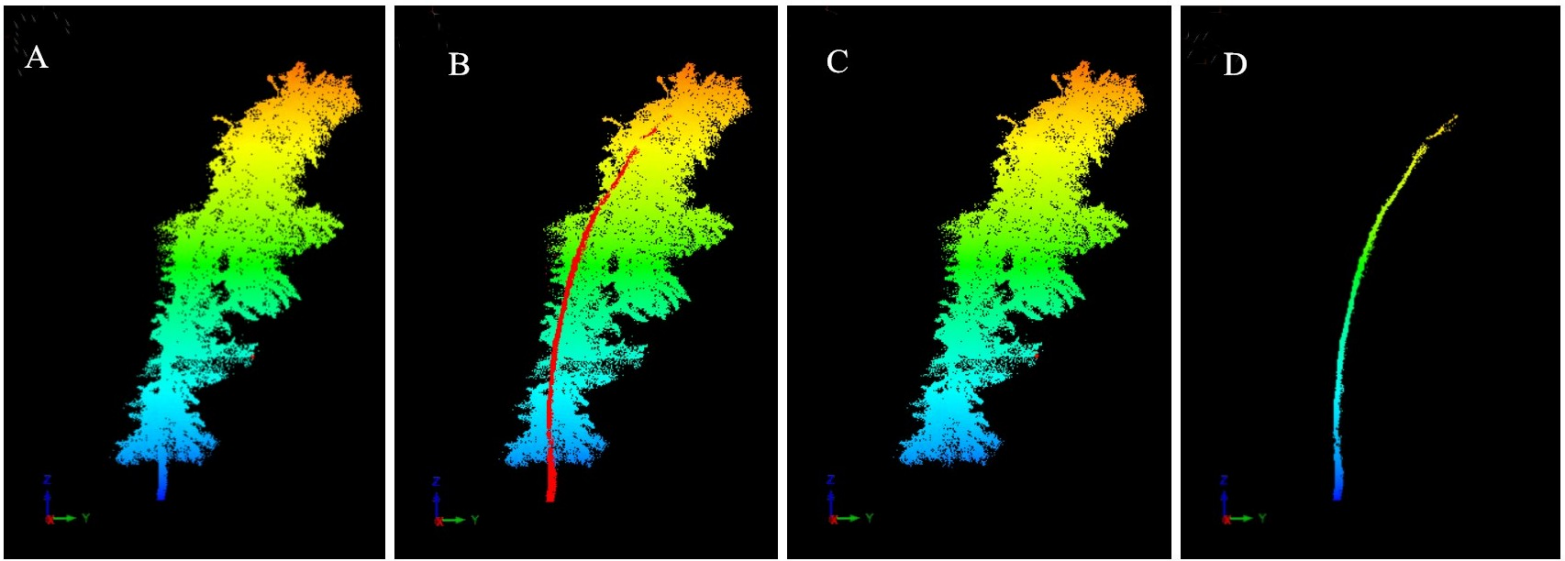
Wood-leaf separation (A: 108230 points; B: 103159 points; C: 5035 points).

The leaf points and wood points were clipped manually under careful visual inspection using RiSCAN PRO 1.64, which is able to rotate and scale the branch point cloud data to better identify leaf points and wood points. We focus on only the first-order branches in this analysis (Fig 4, S2 Fig), and the number of cloud points was counted after wood-leaf separation (Table 3, S2 Table). Seventy percent of the branches were randomly selected as training samples to calculate the ratio of leaf points and wood points to the total points. To assess the above proportions accuracy, 30% of the branches were used as test samples and the relative error was calculated.

**Table 3:** Statistics of the wood-leaf separation results (*L*. *olgensis)*.

	Sample	Mean	Min	Max	Std
Number of total points/(point)	174	78282	7699	475675	72232
Number of leaf points/(point)	174	73639	6524	461067	69960
Number of wood points/(point)	174	4890	248	53758	4837
Relative error/%	49	4.33	0.15	12.02	2.40

### LVV measures of branches

#### LVV measures of branches by 3DS Max

**Fig 5:**
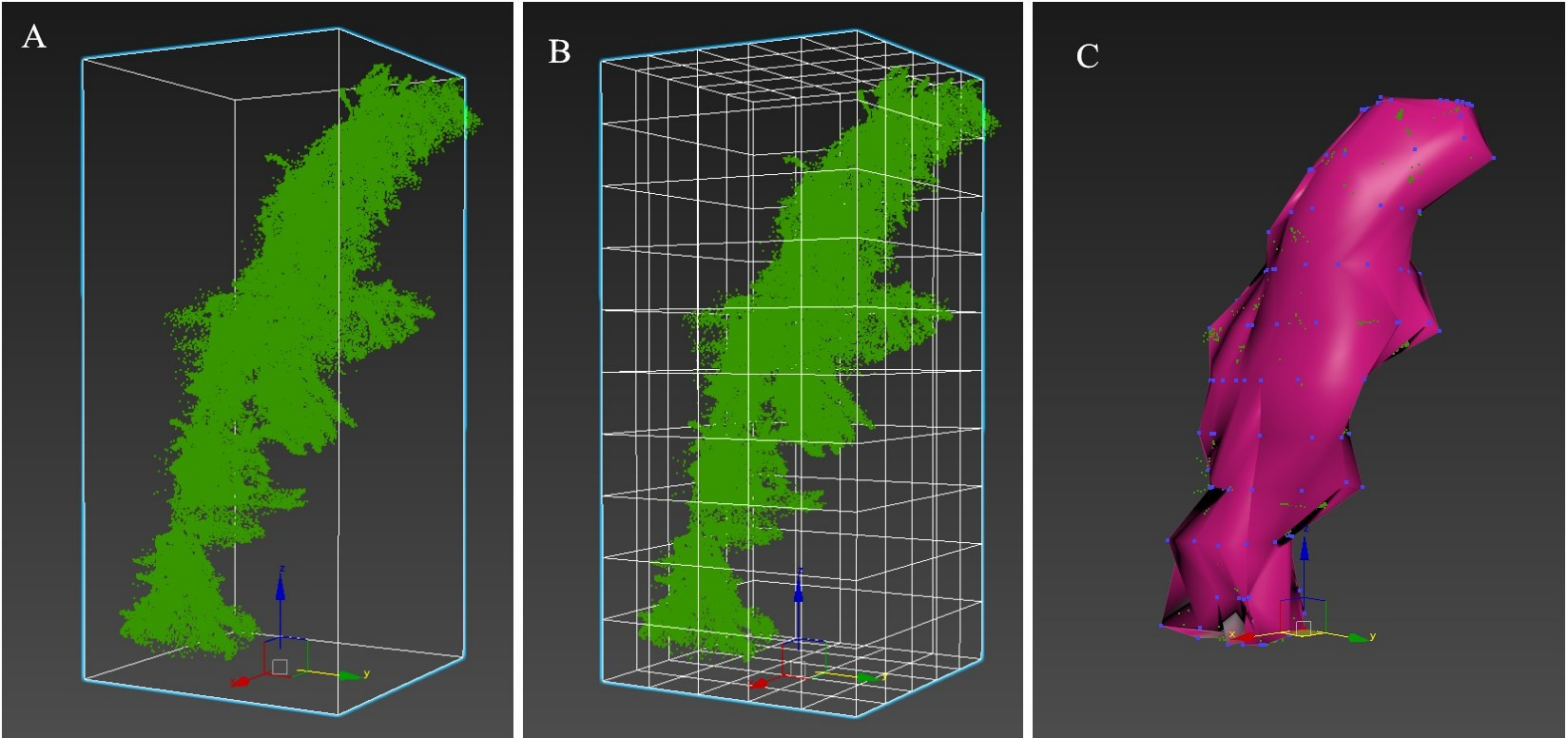
Process of measuring the LVV by 3DS Max topology (A: length and width of the cuboid are 1.3 m, and height is 2.8 m; B: length and width of the cuboid are divided into 5 segments, and the height is divided into 10 segments; C: volume is 1.04 m^3^).

First, the point cloud data are exported to PTS format in RiSCAN PRO 1.64; then, the data in PTS format are imported into Autodesk ReCap, which converts PTS to RCS format, and finally, the RCS format is imported into Autodesk 3DS Max using the point cloud modeler plug-in. In 3DS Max, a standard model was created based on a selected cuboid model rather than a cylinder or sphere. According to the actual branch size, we manually adjusted the length, width and height of the cuboid. We initially set the height of the cuboid to the length of the branch and set the length and width to 1 m to make the cuboid adequately fit the branch outline (for larger and smaller branches, the lengths and widths of the cuboids will increase or decrease correspondingly). The “subsection” parameter information of the cuboid model is modified to form a mesh. The cuboid model is transformed into an editable mesh, and the shapes of branches are established by adjusting the vertexes of the cubes to make the vertexes close to the points on the branches. The results of this method for one branch are shown in Fig 5. Theoretically, the more segments, the more accurate the shape of the branches representation, although the corresponding operation is more complicated. To ensure accuracy and efficiency, we initially divided the length of the cuboid equally into 10 segments, and the width and height were divided into 5 segments (the number of segments were increased or reduced for larger or smaller branches). Finally, the calculation function in the Autodesk 3DS Max software was used to calculate the model volume.

#### LVV measures of branches by triangulated irregular network (TIN)

**Fig 6:**
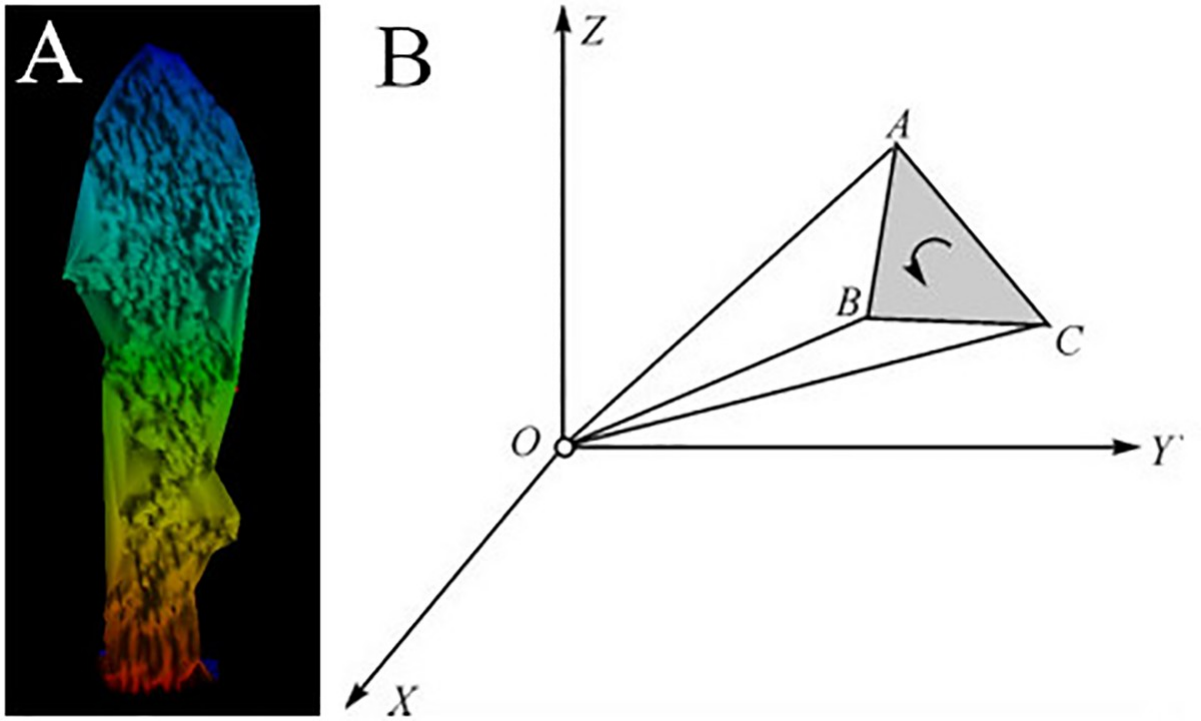
TIN model (A) and tetrahedral volume calculation (B).

In this paper, a spatial triangulated irregular network (TIN) was established by RiSCAN PRO (Fig 6(A)), and then, the volume of the closed TIN was calculated according to the spatial geometry method. Assuming that the three vertex coordinates of any of the triangles are A (*x*_a_, *y*_a_, *z*_a_), B (*x*_b_, *y*_b_, *z*_b_), and C (*x*_c_, *y*_c_, *z*_c_) in the model, the tetrahedral volume is then composed of △ABC as the bottom (ABC counterclockwise order), and the coordinate origin O (0, 0, 0) is the vertex (Fig 6(B)), as shown in formula (1):

$$V_{\mathrm{OABC}}=\frac{1}{6}\left|\begin{array}{ccc} x_{a} & y_{a} & z_{a}\\ x_{b} & y_{b} & z_{b}\\ x_{c} & y_{c} & z_{c} \end{array}\right|$$ (1)

Assuming that the TIN has n triangular patches on its surface, the total volume (*V*_b_) of the closed TIN is given by formula (2):

$$V_{b}=\frac{1}{6}\sum_{i=1}^{n}V_{i}$$ (2)

To verify the accuracy and precision of 3DS Max method, which was used to calculate the volume of the branches, the 3DS Max method and the TIN are compared in this paper because the true values of the branch volumes cannot be obtained. Formula (3) is used to calculate the relative error for comparative analysis.

$$\delta =\frac{\left(V_{0}{‐V}_{i}\right)}{V_{0}}\times 100\%$$ (3)

In the formula, *δ* represent the relative error, *V*_*0*_ represents the LVV calculated from 3DS Max, and *V*_i_ represents the LVV calculated from the TIN.

#### Fill method to estimate LVV

**Fig 7:**
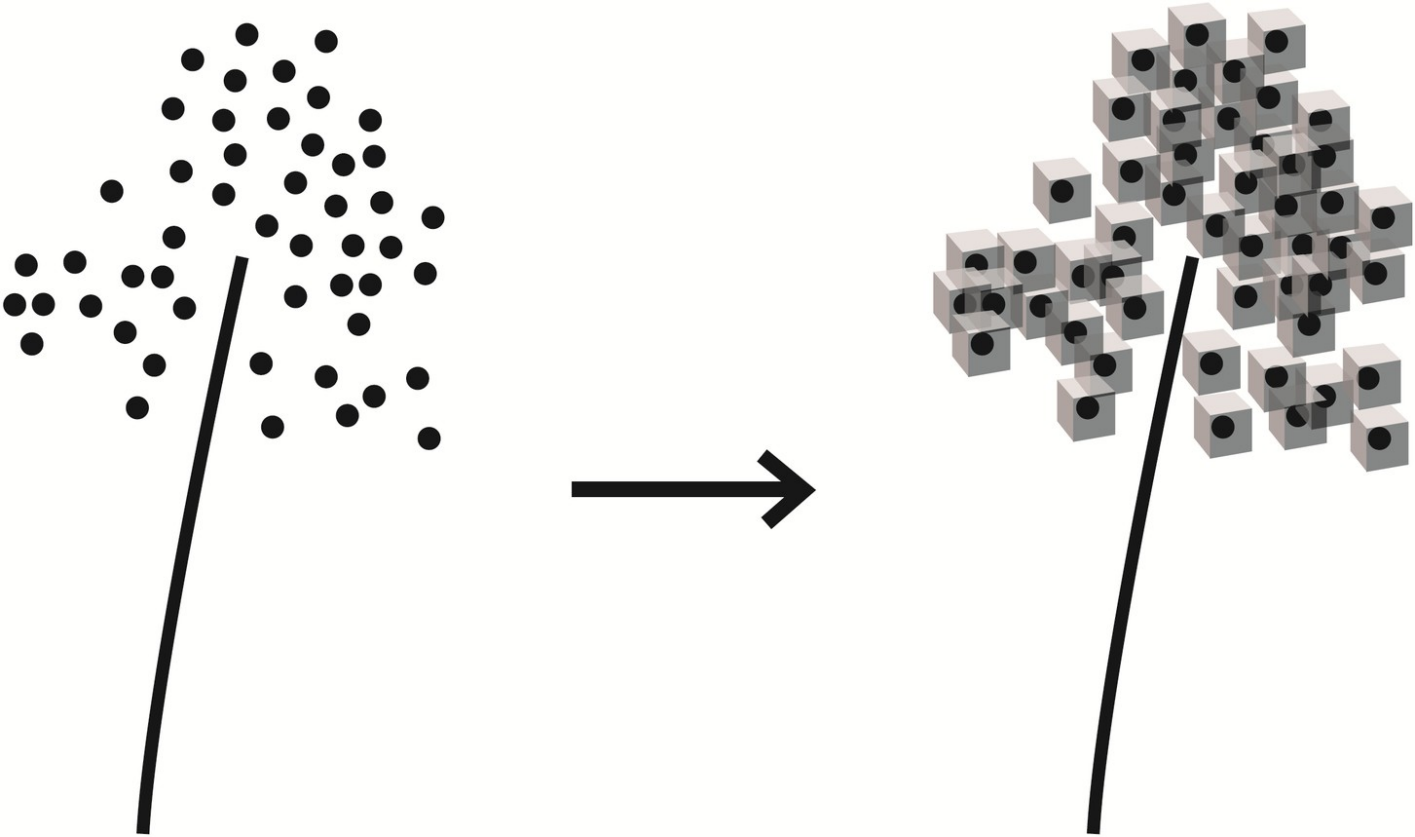
Imaginary fill-type map.

A filling method was to fill discrete point cloud data into entities. Each point in the point cloud data is replaced by a fixed-size cube, and the final LVV of a branch is the sum of several cube volumes, as shown in formula (4):

$$\mathrm{LVV}=V_{1}N+b$$ (4)

(*V*_*1*_: cube size, *N*: the number of leaf points, *b*: constant, Fig 7 is the imaginary fill figure)

In the ideal situation, the constant (*b*) in Eq 4 is 0 and the total volume can be calculated by calculating *V*_*1*_. In this paper, we do not consider other factors that affect the LVV of the branches, such as leaf density, branch length, width, and point density.

### Statistical analysis

We assume that a linear relationship between the number of leaf points and the LVV of a branch appeared using Eq (4). In addition, this relationship was verified by a scatter diagram, drawn by number of leaf points and the LVV of a branch, based on the correlation analysis. Finally, the size of the cube volume was determined further by a regression analysis. The specific steps are as follows.

Step 1: Samples of the 174 *L*. *olgensis* branches were randomly divided into two parts, accounting for 30% and 70% of the total.

Step 2: In SPSS 19.0, 30% of the sample was used to calculate the value of 3DS Max as the dependent variable and the number of leaf points was calculated according to formula (4) as an independent variable in the linear regression that is used calculate the volume of the cube *V*_*1*_.

Step 3: Using 70% of the sample, a linear equation was established between the LVV calculated by the previous step, *V*_*1*_, and the measured value of 3DS Max was used to correct the cube volume to *V*_*1*_.

Step 4: Finally, the LVV is calculated as the estimated value using the corrected volume, *V*_*1*_; the calculated value of TIN is used as the measured value; and the filling method accuracy (C) is used to verify the feasibility of this method. The test formula is as follows.

Residual standard deviation: $S=\sqrt{\frac{\sum {\left(abs-est\right)}^{2}}{n-2}}$, where *abs* and *est* represent the measured and estimated values, respectively; n is the number of samples participating in the accuracy test.

Standard error: $\delta_{x}=\frac{S}{\sqrt{n}}$

Absolute error limit: $\Delta =\delta_{x}\times t_{n-2}^{\alpha },\;where\;t_{n-2}^{\alpha }$ is the t distribution value with confidence levels of 0.05 and 0.01.

Relative error: $E=\frac{\Delta }{X}\times 100\%,\;x=\frac{\sum est}{n}$

Precision: *C = 100%-E*

Meanwhile, we tested the applicability of the filling method with the branches of *Q*. *mongolica*.

## Results

### Wood-leaf separation results

The results of the wood-leaf separation of 174 samples are shown in Table 3. A total of 125 samples are used to calculate the proportions of the leaf points and wood points in branches, and the leaf points and wood points accounted for 91% and 9% of the total points, respectively. In 49 samples, the absolute value of the maximum relative error was 12.02%, the absolute value of the minimum relative error was 0.15%, and the absolute value of the mean relative error was 4.33% (Table 3). The relative error of only one sample was greater than 10%, indicating that we calculated the wood-leaf proportions with a high degree of accuracy. The above wood-leaf proportions was used to calculate the branch point cloud of *Q*. *mongolica*, and the relative error was less than 7% (S2 Table).

### Comparison of LVV measurements by 3DS Max and TIN

**Fig 8:**
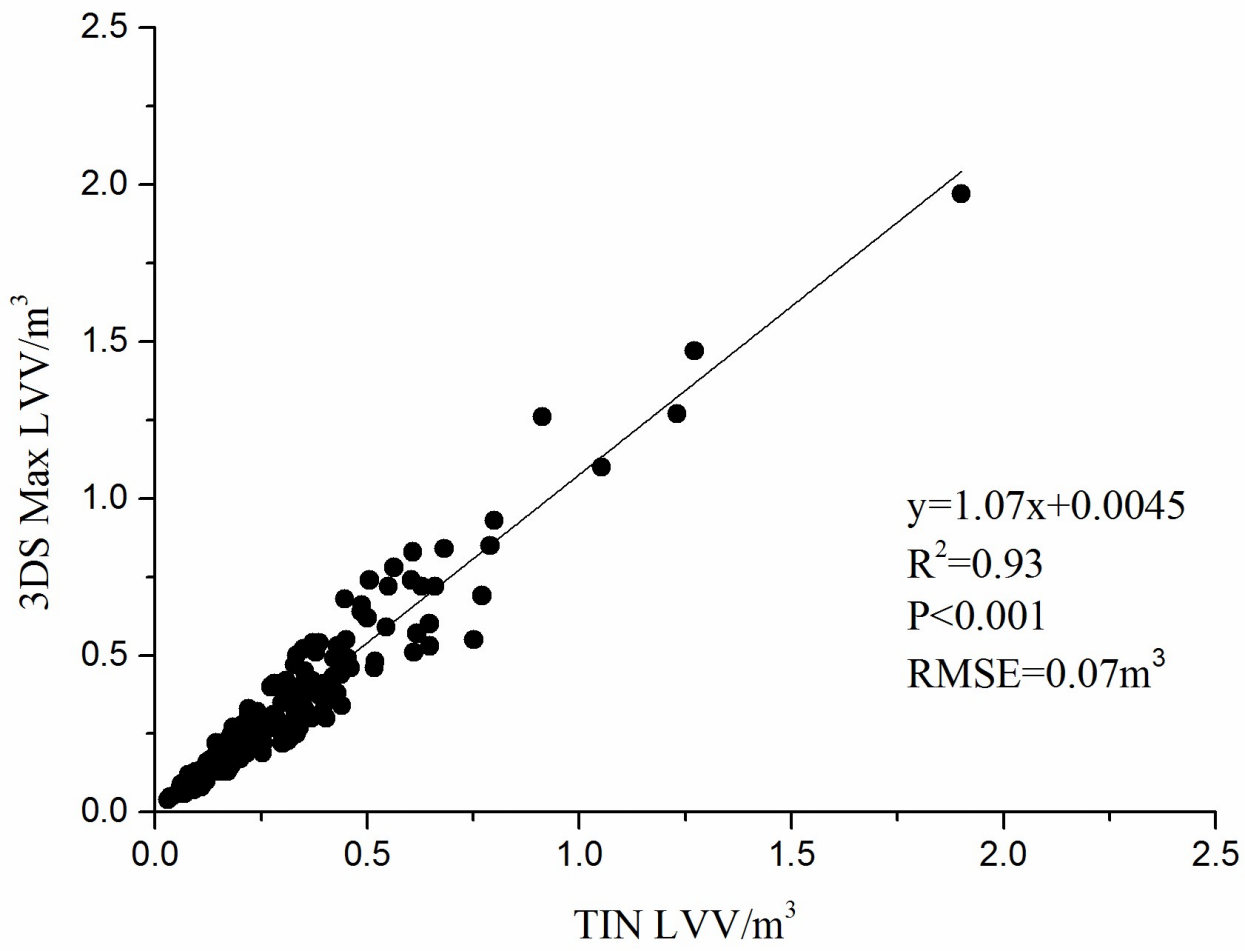
Correlation between the measured value(*L*.*olgensis)*.

**Fig 9:**
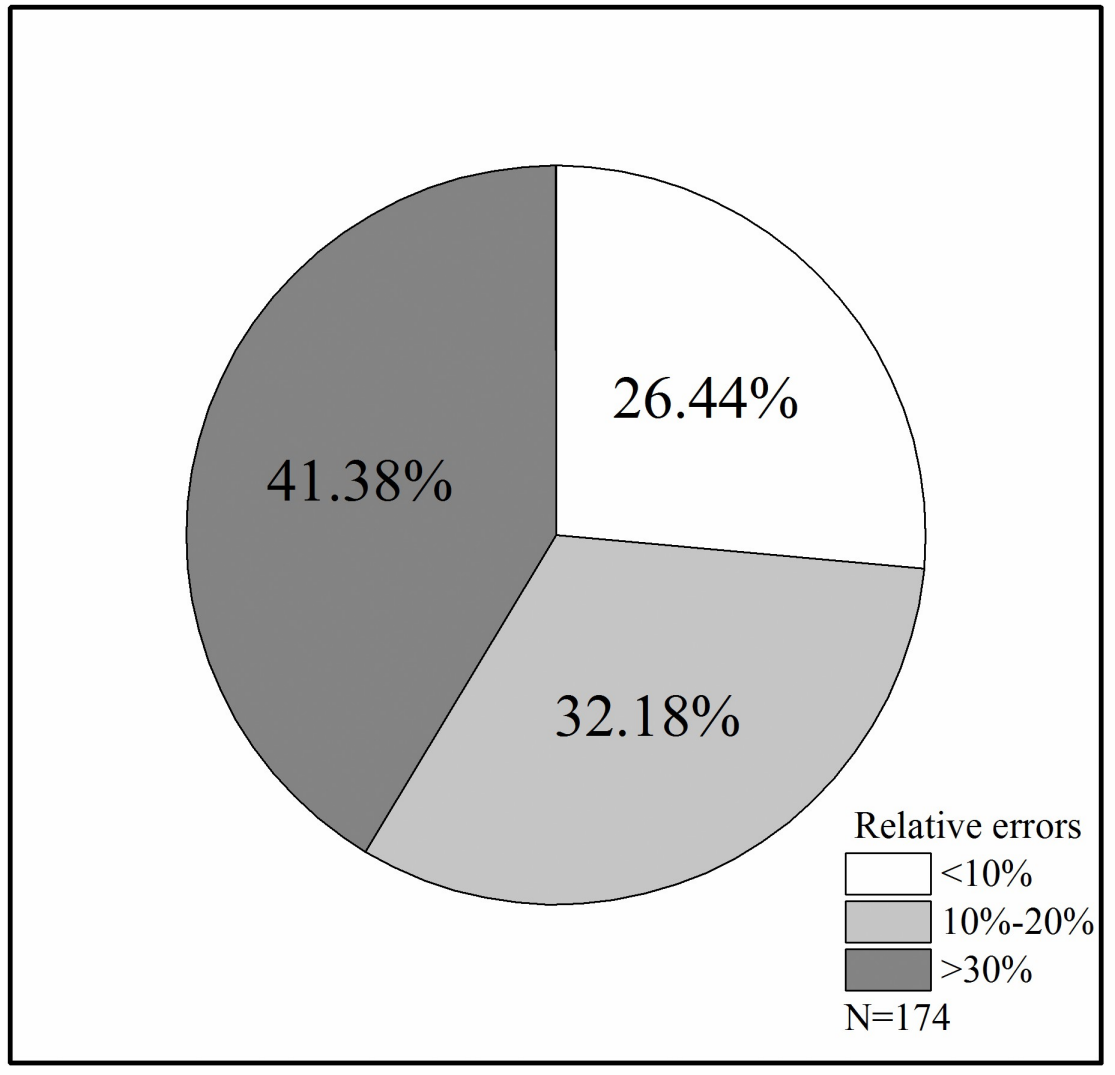
Relative error distribution range of 3DS Max and TIN (*L*. *olgensis)*.

Fig 8 shows the correlation between the values measured by 3DS Max and the values measured by the TIN. It can be seen from the graph that the results of the two methods are consistent to a certain extent (R^2^ = 0.93, P<0.001, RMSE = 0.07 m^3^). The relative errors of the results from the two methods were further calculated, revealing that the absolute value of the maximum relative error was 37.12%, the absolute value of the minimum relative error was 0.03%, and the absolute value of the average relative error was 17.70% (Table 4). Fig 9 shows the distribution of the relative errors of 174 samples; the relative errors of 72 samples were greater than 30%, those of 56 samples were between 10% and 20%, and those of 46 samples were less than 10%, indicating that in some samples, the results of the two measurement methods exhibit large deviations. The results of the branches of *Q*. *mongolica* were shown in S2 Fig, and the average relative error was 23.15% (S3 Table). When using 3DS Max to measure the volume of branches, the shape of the branches can be manually approximated, but there are inevitably errors. The TIN must use the entity to calculate the volume, and the current technology used to transform the multifaceted grid to the entity is not yet mature. When the closed TIN is constructed in RiSCAN PRO, the system exhibits some deviation from the recognition of the surface point. By contrast, the 3DS Max visually represents better the shape of the branch. Therefore, the difference of the TIN is greater than that of the 3DS Max. As the current real values of the branch volumes cannot be obtained, we can only conduct a relative analysis.

**Table 4:** Statistics of the LVV measurement results (*L*.*olgensis)*.

Method	Sample	Mean	Min	Max	Std
3DS Max/(m^3^)	174	0.34	0.04	1.97	0.27
Triangulated irregular network /(m^3^)	174	0.31	0.03	1.90	0.24
Relative error/%	174	17.70	0.03	37.12	10.04

### Analysis of correlation between LVV and number of leaf points

**Fig 10:**
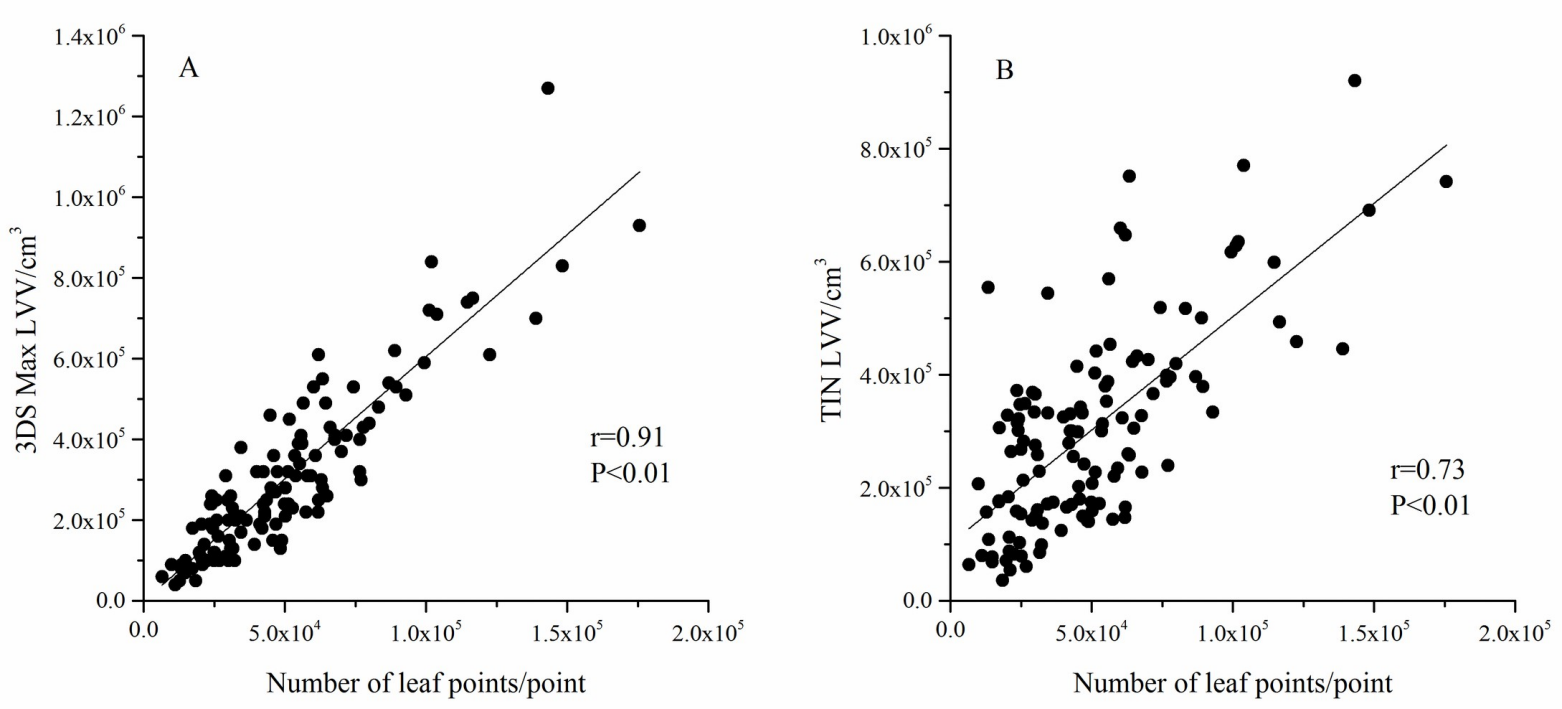
Correlation between LVV and leaf point number in branch (*L*.*olgensis)*.

Fig 10(A) shows that there is a linear relationship between the LVV value measured by 3DS Max and the number of leaf points, preliminarily proving that our hypothesis is true. However, the correlation between the LVV value measured by the TIN and the number of leaf points was significantly reduced (Fig 10(B)). The correlation coefficient (r) decreased from 0.91 to 0.73 (*P*<0.01). The same trend was seen in the branches of *Q*. *mongolica* (S3 Fig).

### Filling method to measure LVV

**Fig 11:**
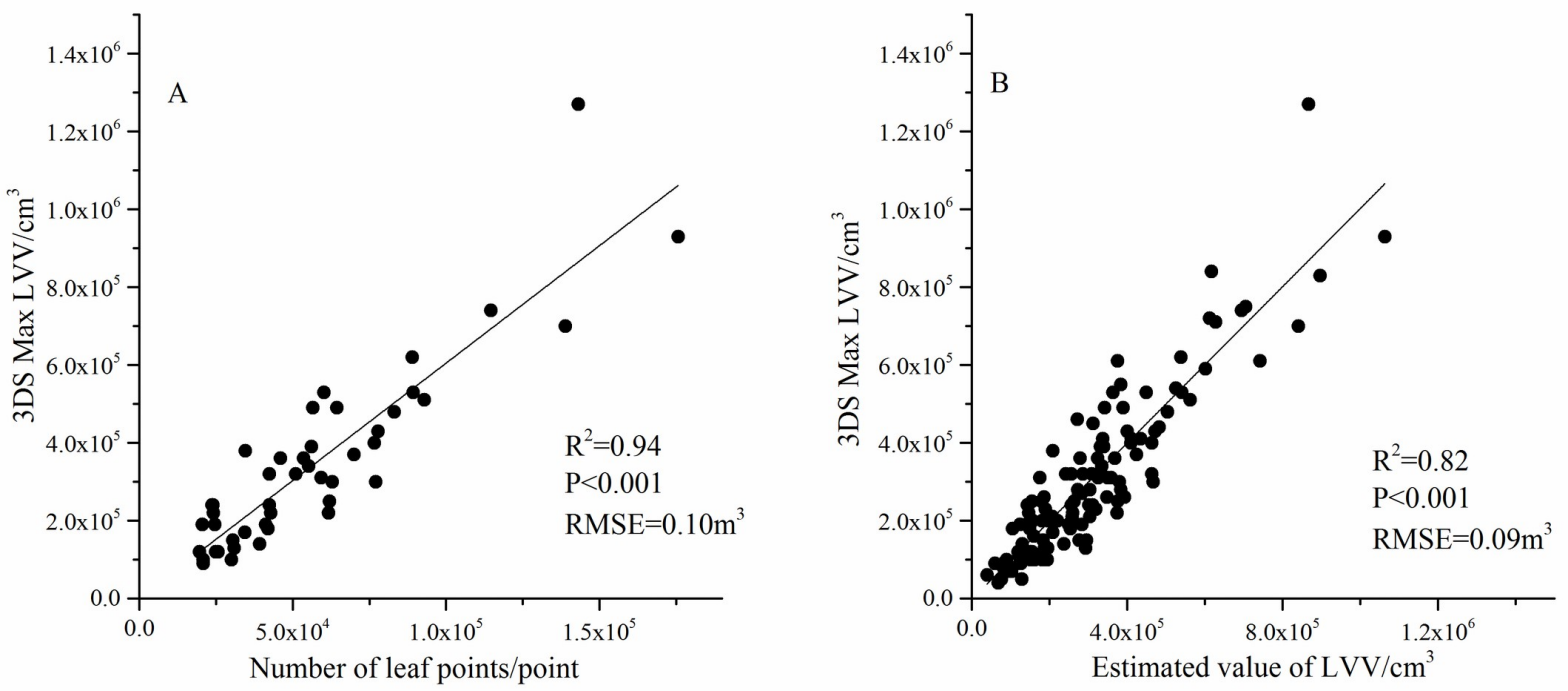
Cube volume calculation (A) and volume correction (B).

First, a linear regression of 30% of the samples was used to obtain Eq 1 (*LVV* = 6.03*N*+1430.79, *R*^*2*^ = 0.81, *P*<0.001, *RMSE =* 0.10 m^3^). The coefficient of determination (*R*^*2*^) of the regression equation was high and passed the F test (*P* <0.001). This result shows that there is a significant linear relationship between the number of leaf points and the LVV of the branches. However, the constant of Eq 1 is not significant, and the intercept of the linear equation is 0 and was refitted to obtain Eq 2 (*LVV* = 6.05*N*, *R*^2^ = 0.94, *P* <0.001, *RMSE =* 0.10 m^3^; Fig 11(A)). Then, 70% of the sample was used to estimate the LVV of the branches by Eq 2, and regression Eq 3 (*y* = 1.004*x*-1970.66, *R*^*2*^ = 0.82, *P*<0.001, *RMSE* = 0.09 m^3^; Fig 11(B)) was established between the measured values. Finally, Eq 3 was used to correct the cube volume *V*_*1*_ = 6.11 cm^3^.

### Accuracy of the filling method

By calculating the accuracy of the LVV, the results show that the accuracies of the LVV of the *L*. *olgensis* branches were 94.20% and 91.78% at the 95% and 99% confidence levels, respectively (Table 5). In addition, the proportions of the leaf points (91%) was then used to calculate the number of leaf points of the samples involved in the test, and the LVV measurement accuracy was recalculated. The measuring precision of LVV was 94.35% and 91.99% at the 95% and 99% confidence levels, respectively (Table 5). In addition, the measuring accuracy of the branches in *Q*. *mongolica* was 90.01% and 85.63% (S4 Table).

**Table 5:** Testing results of the LVV measurements by the filling method (*L*. *olgensis)*.

Item	Calculated as the number of manually separated leaf points		Calculated as the proportion to the number of leaf points	
	α = 0.05	α = 0.01	α = 0.05	α = 0.01
Sample size	174		174	
Measured value/cm^3^				
Total	50340507.00		50340507.00	
Mean	299072.29		299072.29	
Estimated value /cm^3^				
Total	51424277.51		50448927.94	
Mean	307911.80		302089.39	
Residual Standard deviation (*S*)	139107.32		132954.45	
Standard error (*δ*_*x*_)	10764.45		10288.32	
$t_{n‐2}^{\alpha }$	1.658	2.351	1.658	2.351
Absolute error limit (Δ)	17847.45	25307.22	17058.04	24187.85
Relative error (*E/*%)	5.80	8.22	5.65	8.01
Precision (*C/*%)	94.20	91.78	94.35	91.99

Finally, it may be concluded that the equation for calculating the LVV based on the leaf point cloud distribution is “*LVV* = 6.11*N*”. After using T-LiDAR to acquire point cloud data in the field, the number of point clouds is the easiest parameter to obtain. Therefore, use of this equation would enable researchers and investigators to quickly estimate the LVV of the branches. The character of this work is explorative, as there is no predefined set of hypotheses or theoretical framework to test. Rather, we would like to contribute to the formation of hypotheses as a background for further studies.

## Discussion

### Wood-leaf separation

In this study, we used RiSCAN PRO 1.64 to manually separate the leaf points and wood points from the first-order branches and calculate the ratio of the leaf points and the wood points to the number of point clouds. Careful visual inspection indicated that manual cutting results were detected with high accuracy. Existing approaches have used intensity information [41, 42], a multiwavelength approach [43], or waveform information [44] for wood-leaf separation. Successful intensity-based wood-leaf separation using one LiDAR system does not guarantee successful separation using other LiDAR systems [40]. The development of multiwavelength LiDAR is still in the early stages [45]. Recently, a geometric approach for wood-leaf separation was reported [46, 47], although this method applies to broad-leaved trees. To the best of our knowledge, the wood-leaf separation algorithms based on terrestrial point cloud data are technologically challenging, and there is no widely adopted method. With current technology, the use of the manual method for wood-leaf separation is still reliable under careful visual inspection. In this paper, *L*. *olgensis branches* were used to calculate the wood-leaf ratio of a point cloud was also applicable to *Q*. *mongolica*. It is preliminarily proved that coniferous species and broad-leaf species have the same wood-leaf ratio of a point cloud under the same scanning setting. This ratio can be used as the basis for verifying the algorithm of wood-leaf segmentation and has great practical significance. In future studies, it is still necessary to explore the wood-leaf ratio of different scanning instruments.

### Measurement of LVV

The measurement of the LVV of a tree is essentially the measurement of the volume of the tree crown (or the volume of the branches). However, obtaining the precise volume of a crown is a challenging task because of the complexity of the tree structure. In this paper, we divide point cloud data from branches into leaf points and wood points and propose a method to measure the LVV based on the leaf point cloud distribution. The filling method is used to calculate the volume of the whole by filling a geometric body of a fixed size from the inside of a three-dimensional model. Accuracy analyses carried out on all branches confirmed a good performance of filling method that can quickly provide estimates for a large number of samples. This paper only proves that the filling method is feasible, and our aim is to provide a new feasible idea for the calculation of three-dimensional green space.

**Fig 12:**
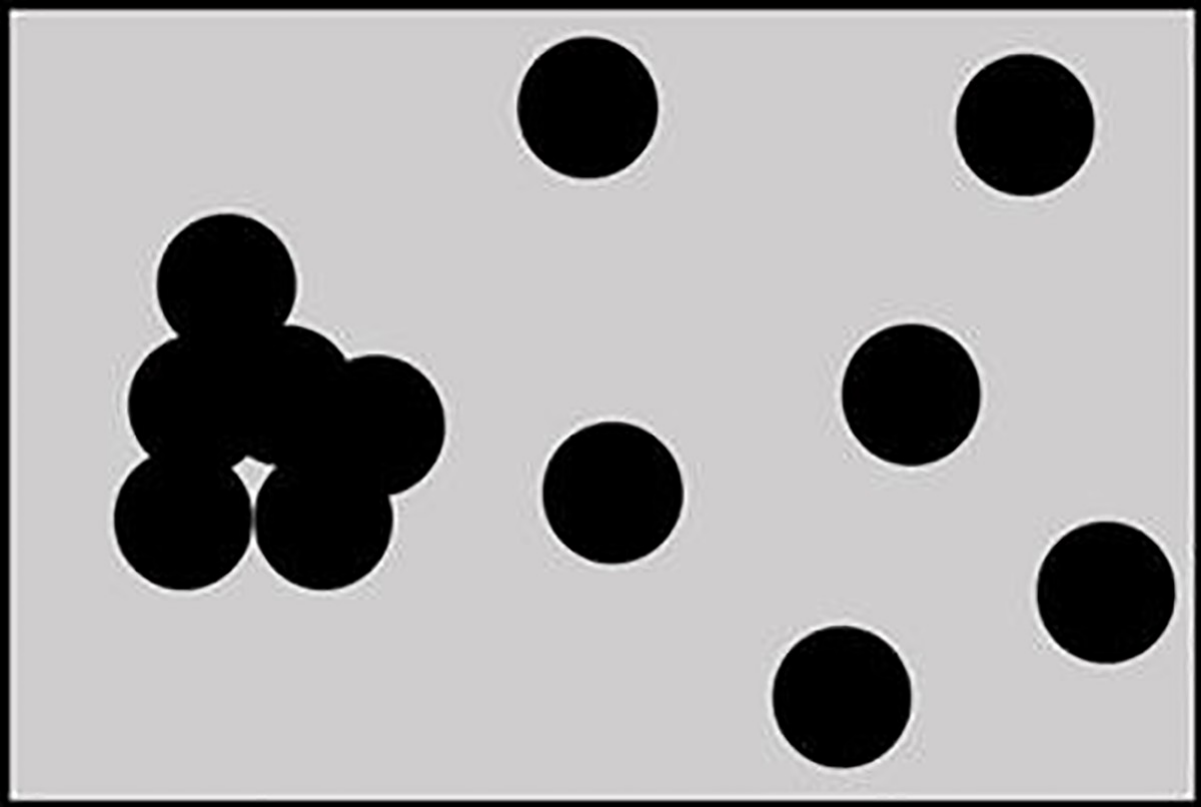
Random distribution of possible internal points in branches.

The filling method is a simple and direct method. According to our equation, the LVV is only related to the number of point clouds. The results show that although the method has good performance, other factors that affect the three-dimensional green space of branches are not considered, such as leaf density, branch length (*L*), width and point density. For example, two similar branches should have similar LVVs in real situations even if the internal points are largely different. We originally envisioned using the same size cube to fill the branch space in an ideal situation because we obtained a good correlation. In fact, due to the reflection of the laser beam between branches and leaves, the obtained internal points were discrete. After the cube was used to replace the points, some points overlapped, while some were far away (Fig 12). In addition, the points on the surface should fall at the boundary of the 3D convex hull, and they should not be filled with the cube similar to the points in the interior. Because we cannot separate out the points on the surface, our equation includes the surface points. This phenomenon is also one of the uncertainties. At the same time, branches with different canopy heights have different leaf densities, and the sizes of the filled cubes may be different. Therefore, no absolute conclusion can be drawn about the effectiveness of the filling method itself.

**Fig 13:**
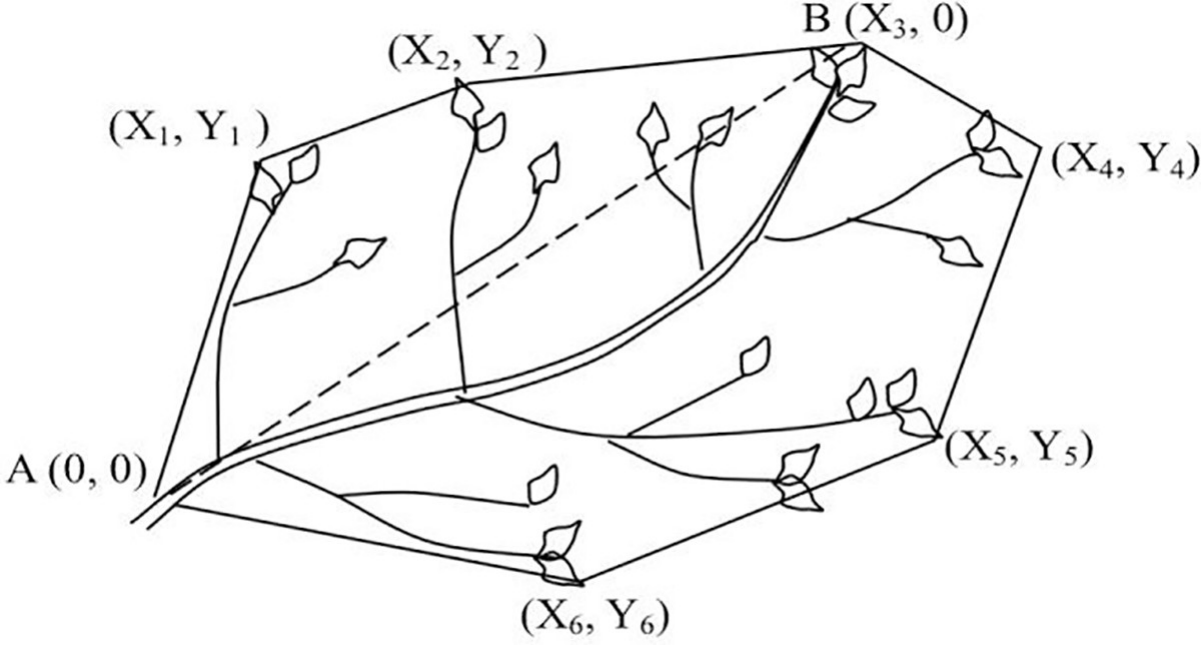
Conventional measurement methods of the branch volume (A: needle closest to the tree stem, B: tip of the needle farthest from the stem. The dashed line depicts the horizontal axis.) (Image from [48]).

**Fig 14:**
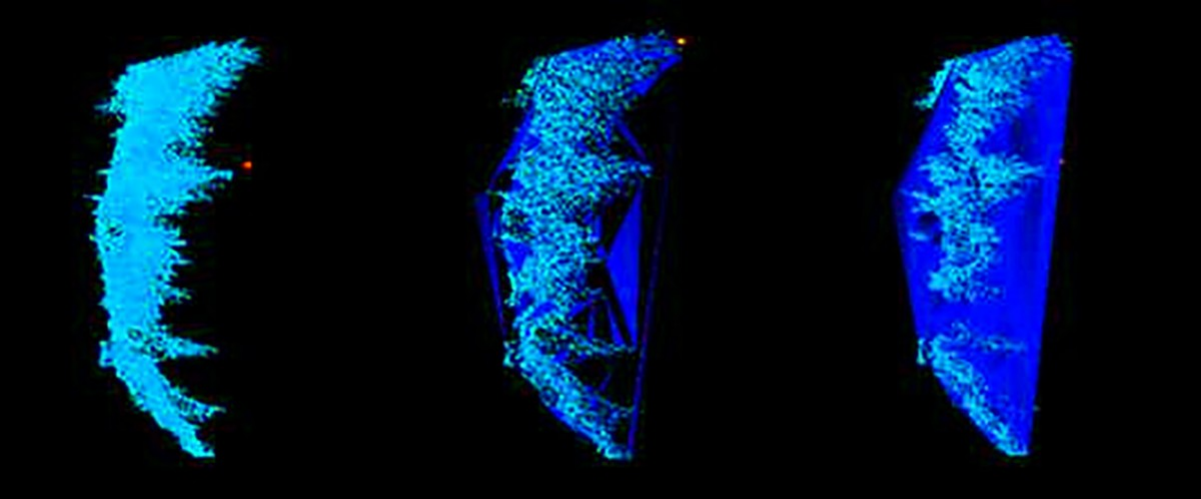
Error diagram of triangulated irregular network measurement and calculation.

Zeide [48] defined branch volume as the smallest convex hull that envelops all of the space with foliage growing on a given branch. Aa shown in Fig 13, the volume occupied by the foliage of a given branch was estimated as the product of the area of the convex polygon that circumscribes the foliage and the foliage depth in the direction perpendicular to the polygon. This method is equivalent to using a simple polyhedron instead of the branch volume. In fact, this definition leads to the inclusion of the space that is not occupied by foliage. In terms of the crown volume measurement, its core idea is to convert the crown into an entity and then calculate the volume of the entity. For example, Kato *et al*. [49] proposed a “wrapped surface reconstruction” method. In addition, the key principles of the 3D and triangulated irregular network used in this paper are similar, and the point cloud data form the surface points are used to create the convex hull. Using the point cloud data to build the spatial triangulated irregular network and calculate the volume of the model according to the spatial geometry, this method is difficult because the volume must be estimated by an approximate polyhedron. The higher the number of polyhedrons, the higher the accuracy. Software can accurately approach the surface of the object with polyhedral mesh when simulating the object, although the computational volume must be solid, and there is no mature module for the transformation between a polyhedral mesh and an entity. When we used RiSCAN PRO 1.64 to construct a closed triangulated irregular network, we found that the system could not accurately identify the surface points, and the surface points identified by the system formed a spatial tetrahedron with any of the surrounding points (Fig 14). We used 3DS Max to manually establish the convex hull of the branch point cloud data, and the results of the volume measurements were closer to the actual situation of the branch than the results from the conventional method. The method of manually establishing the convex hull can approximate the contour of the branch to the maximum extent, avoiding the error of the triangulated irregular network to the surface point recognition. However, the artificial manual topology will inevitably result in errors, and the disadvantage of this method is that it cannot be automatically processed, and the accuracy of its measurement depends on the multifaceted grid settings. Moreover, the accuracy is higher when there are more grids, although the corresponding operation is more cumbersome. Approximately 20 to 30 minutes is required to manually build a branch topology; therefore, the method is not suitable for calculating the LVV at large scales (canopy or stand). In contrast, the filling method is a simple and straightforward approach. However, even if our approach could be applied without distinguishing tree species and tree shape, the size of the cube is also affected by some uncertain factors. Any changes in scanning instrument and/or scanning setup (e.g., number of scans, distance away from tree, etc.) could potentially affect the point cloud density and complexity and consequently the selection of geometric size.

The crown is not a three-dimensional solid. The real canopy and each branch contain many spaces inside, and the collection is a multilevel structure. From the concept of the LVV, there is no biomass (leaf and stem) in the gap between branches and leaves. To be exact, the true value of three-dimensional green space can only be obtained by removing the empty parts from the volume of the crown or branch. Unfortunately, these gaps cannot be removed due to technical limitations. All we can do for volume measurements is minimize the differences in the gap estimates from the actual situation. The study of LVV is based on the ecological function and the physiological metabolism characteristics of plant stems and leaves, which reflect the material flow, energy conversion and utilization of plants. As the most important organ used to capture solar radiation, absorb carbon dioxide and release oxygen in terrestrial ecosystems [50], the sizes of plant leaves are significant. We refer to the size of the space occupied by the blade as the “effective LVV”. The LVV is a new research topic, and different plants have different spatial structures. The specific manifestation is that the total amount of leaves and the size of the space occupied by the leaves are different, so the scientific study of three-dimensional green space can better reveal the ecological role of plants.

### Problem of terrestrial point cloud data

**Fig 15:**
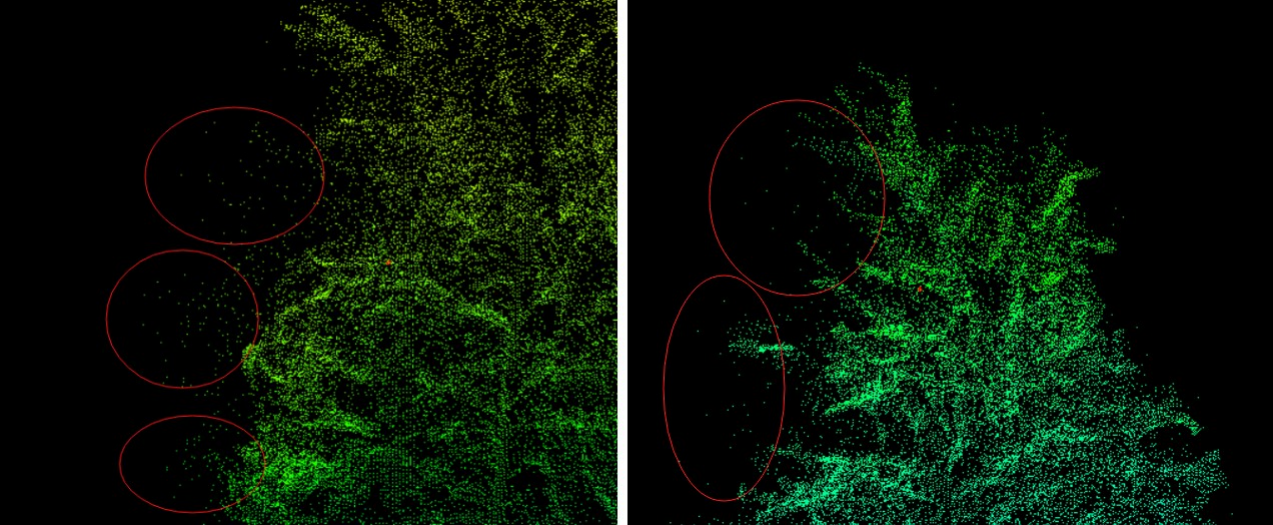
Local magnification of branch point clouds.

The description quality of within-stand scanned trees is highly dependent on the occlusion level caused by surrounding trees and wind. Occlusion causes discontinuity in the description of the tree axes, whereas wind induces wave-shaped axes and increases noise in terrestrial point cloud data [51]. We used five scanning positions to enhance the integrity of the point cloud data, but multiple scans can result in a large amount of point cloud data and redundancy, making it difficult for the computer to operate. Although the point cloud data are denoised before they are used, there is still noise in the data. Fig 15 presents a local magnification diagram of the branch point cloud data, and some points at the edge obviously do not belong to the branch, and these points need to be artificially determined and removed. However, some points are uncertain, which is subjective.

The research of parameter extraction based on T-LiDAR includes crown volume, wood volume, H, and DBH; i.e., accuracy is the focus of attention and depends on the accuracy of the data source and the accuracy of the reference measurements themselves [52]. For the calculation of LVV, the lack of fine-sampling manual measurements is often an important constraint [26]. Given the complexity of plant structures and forest environments, the use of T-LiDAR in forest environments remains a challenge, especially for forestry applications. It is necessary to develop a point cloud data processing platform for forestry applications, which would help promote the application of LiDAR in forestry.

## Conclusions

The objective of the present research was to introduce a filling method for LVV measurements and verify the feasibility of the method by using terrestrial point cloud data from *L*. *olgensis* and *Q*. *mongolica* branches. In the proposed method, the branch point clouds are divided into leaf points and wood points, and then the equation (*LVV = V*_*1*_*N*, where *N* is the number of leaf points, and *V*_*1*_ is cube size) is used to calculate LVV. In this study, we used RiSCAN PRO 1.64 to manually separate the leaf points and wood points under careful visual inspection and calculated that leaf points and wood points accounted for 91% and 9% of the number of the point clouds of branches. This wood-leaf ratio of a point cloud applies to both *L*. *olgensis* and *Q*. *mongolica* branches. We found that when the laser transmitting frequency was 300,000 points/second and the point cloud dilution utilized the octree method, the cube (*V*_*1*_) size was 6.11 cm^3^. Our method performed well for coniferous species and broad-leaf species. A comparison of the results obtained using our method and those obtained using the TIN approach suggested that our method is superior. As tested, the measuring accuracy of *L*. *olgensis* and *Q*. *mongolica* at the levels of α = 0.05 and α = 0.01 are (94.35%, 90.01%) and (91.99%, 85.63%) respectively. However, even if our approach could be applied without distinguishing tree species and tree shape, the size of the cube is also affected by uncertain factors (e.g., scan setup, distance from tree, level of details acquired by TLS, etc.). Finally, the results achieved in this study represent a good starting point for measuring LVV from terrestrial point cloud data, although the proposed approach was only tested in a branch point cloud under the same scanning setting. Further studies are consequently still needed to evaluate the applicability of the method in different forest stands and/or different scan setups.

## Supporting information

S1 Fig Point cloud of the *Q*. *mongolica* branch. (TIF)

S2 Fig Correlation between the measured value (*Q*. *mongolica*). (TIF)

S3 Fig Correlation between the LVV and leaf point number of a branch (*Q*.*mongolica*). (TIF)

S1 Table Basic information of *Q*. *mongolica* branches. (DOCX)

S2 Table Statistics of the wood-leaf separation results (*Q*. *mongolica*). (DOCX)

S3 Table Statistics of the LVV measurement results (*Q*. *mongolica*). (DOCX)

S4 Table Testing results of measurements of the LVV by the filling method (*Q*. *mongolica*). (DOCX)
