# Supplementary figures and images for: A new approach for estimating living vegetation volume based on terrestrial point cloud data
Source: PLoS One. 2019 Aug 29;14(8):e0221734. doi: 10.1371/journal.pone.0221734 (PMC6715214; doi:10.1371/journal.pone.0221734)

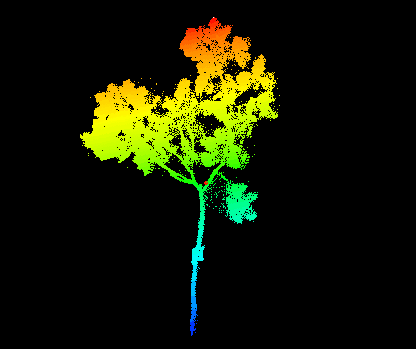

Supplement: S1 Fig — (TIF) [file pone.0221734.s001.tif]

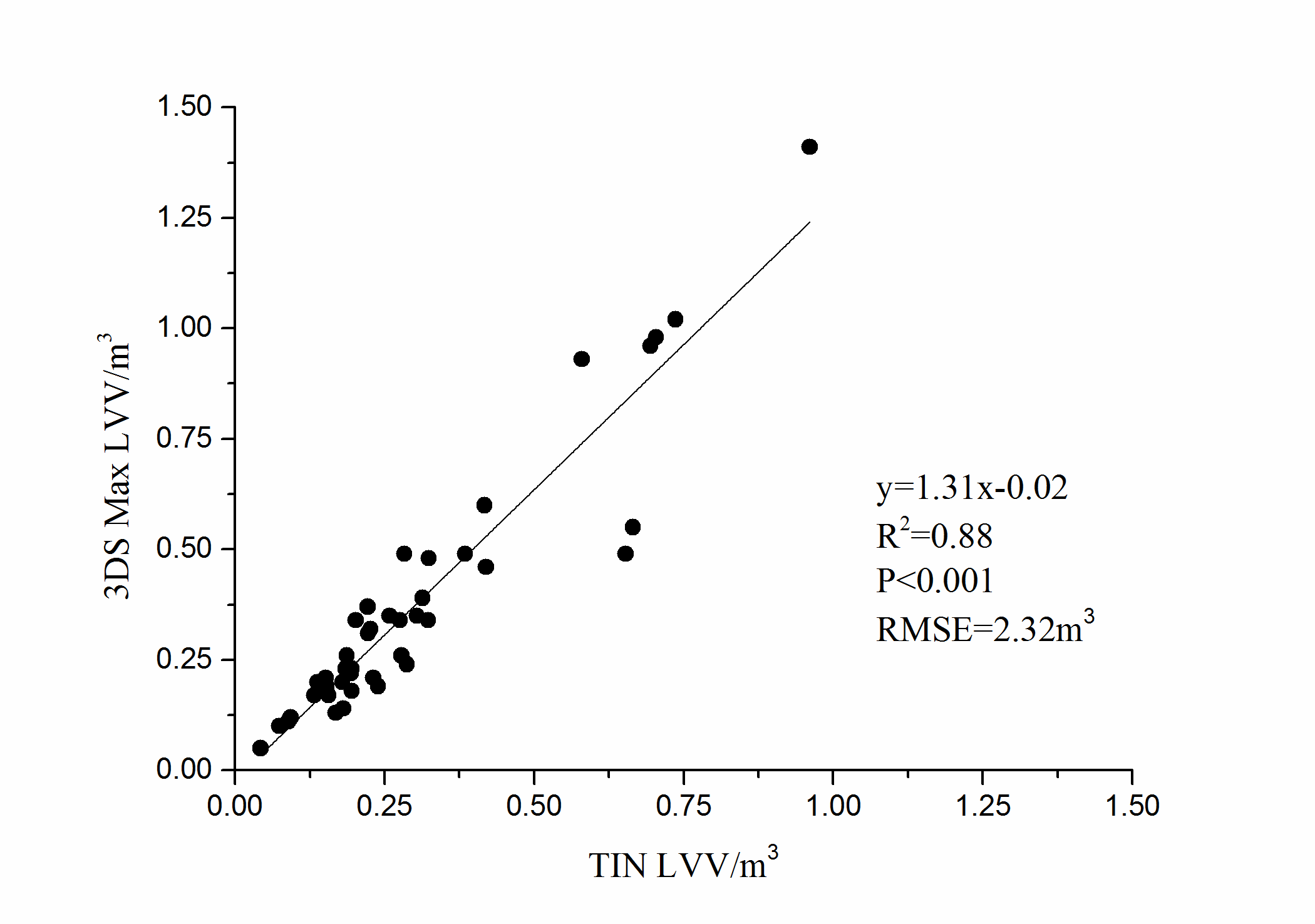

Supplement: S2 Fig — (TIF) [file pone.0221734.s002.tif]

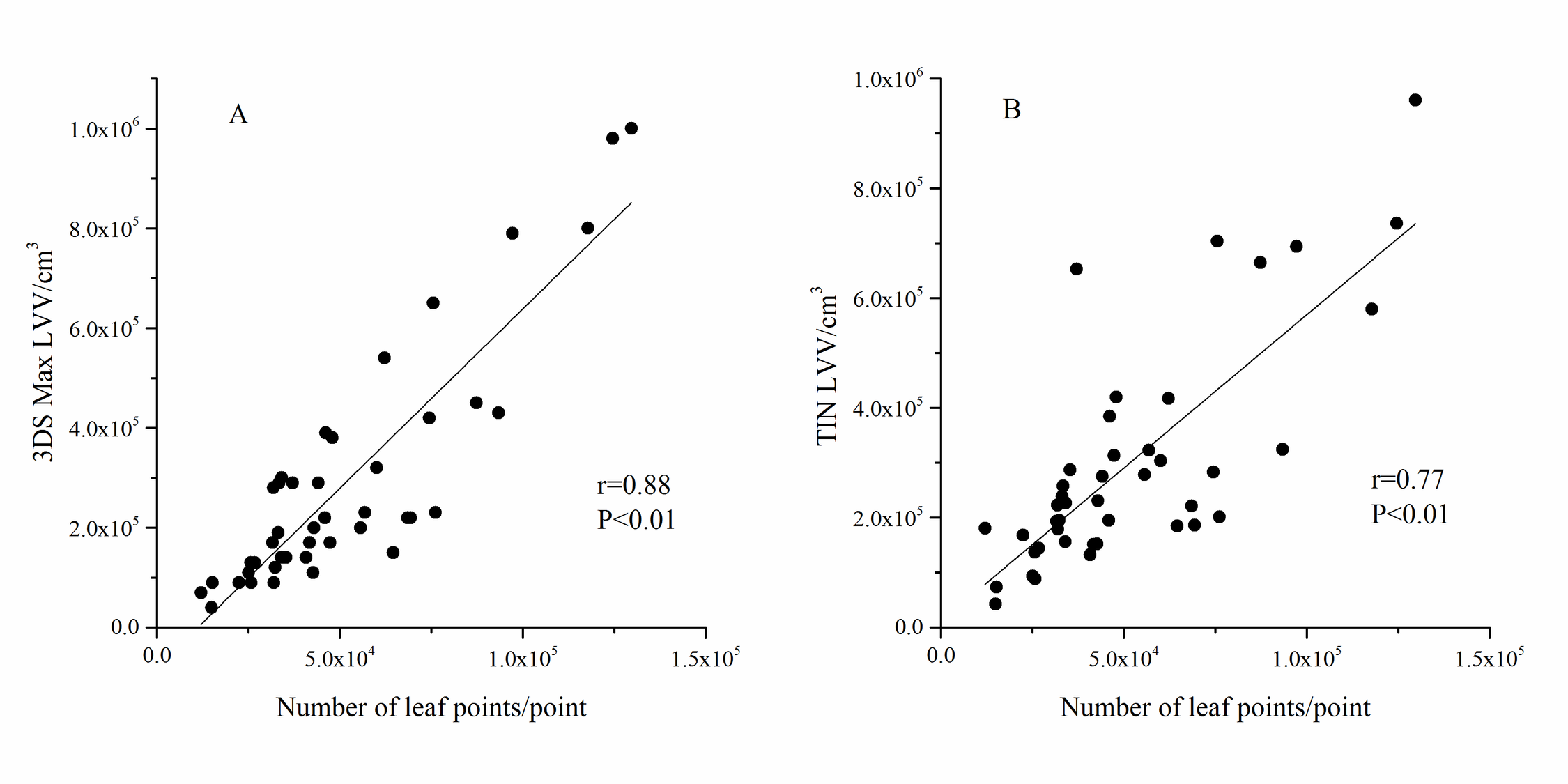

Supplement: S3 Fig — (TIF) [file pone.0221734.s003.tif]
