# Supplementary material for: A new approach for estimating living vegetation volume based on terrestrial point cloud data
Source: PLoS One. 2019 Aug 29;14(8):e0221734. doi: 10.1371/journal.pone.0221734 (PMC6715214; doi:10.1371/journal.pone.0221734)
Supplement: S1 Table — (DOCX) [file pone.0221734.s004.docx]

| Branch factors | Mean | Min | Max | Std |
| --- | --- | --- | --- | --- |
| *d*/(mm) | 28.10 | 12.02 | 50.56 | 9.75 |
| *L*/(cm) | 176.95 | 50 | 300 | 55.20 |

Note: *d*: diameter of the branches, *L*: length of the branches
