# Supplementary material for: A new approach for estimating living vegetation volume based on terrestrial point cloud data
Source: PLoS One. 2019 Aug 29;14(8):e0221734. doi: 10.1371/journal.pone.0221734 (PMC6715214; doi:10.1371/journal.pone.0221734)
Supplement: S2 Table — (DOCX) [file pone.0221734.s005.docx]

|  | Sample | Mean | Min | Max | Std |
| --- | --- | --- | --- | --- | --- |
| Number of total points/(point) | 43 | 55594 | 13954 | 141805 | 30732.35 |
| Number of leaf points/(point) | 43 | 51491 | 11995 | 129670 | 28946.67 |
| Number of wood points/(point) | 43 | 4105 | 895 | 12135 | 2433.25 |
| Relative error/% | 43 | 2.91 | 0.24 | 6.75 | 1.83 |
