# Supplementary material for: A new approach for estimating living vegetation volume based on terrestrial point cloud data
Source: PLoS One. 2019 Aug 29;14(8):e0221734. doi: 10.1371/journal.pone.0221734 (PMC6715214; doi:10.1371/journal.pone.0221734)
Supplement: S3 Table — (DOCX) [file pone.0221734.s006.docx]

| Method | Sample | Mean | Min | Max | Std |
| --- | --- | --- | --- | --- | --- |
| 3DS Max/(m^3^) | 43 | 0.37 | 0.05 | 1.41 | 0.29 |
| Triangulated irregular network /(m^3^) | 43 | 0.30 | 0.04 | 0.96 | 0.21 |
| Relative error/% | 43 | 23.15 | 5.13 | 42.24 | 9.58 |
