# Supplementary material for: A new approach for estimating living vegetation volume based on terrestrial point cloud data
Source: PLoS One. 2019 Aug 29;14(8):e0221734. doi: 10.1371/journal.pone.0221734 (PMC6715214; doi:10.1371/journal.pone.0221734)
Supplement: S4 Table — (DOCX) [file pone.0221734.s007.docx]

| Item | Calculated in proportion to the number of leaf points | |
| --- | --- | --- |
|  | α=0.05 | α=0.01 |
| Sample size | 43 | 43 |
| Measured value/cm^3^ | 12460000.00 | |
| Total |  |  |
| Mean | 289767.40 | |
| Estimated value /cm^3^ |  |  |
| Total | 13450791.15 | |
| Mean | 312809.10 | |
| Residual Standard deviation (*S*) | 121626.52 | |
| Standard error ($\text{δ}_{\text{x}}$) | 18547.87 | |
| $\text{t}_{\text{n-2}}^{\text{α}}$ | 1.684 | 2.423 |
| Absolute error limit ($\Delta$) | 31234.61 | 44941.49 |
| Relative error (*E/*%) | 9.99 | 14.37 |
| Precision (*C/*%) | 90.01 | 85.63 |
